# Novel inhibitory effect of galectin-3 on the respiratory burst induced by *Staphylococcus aureus* in human neutrophils

**DOI:** 10.1093/glycob/cwad032

**Published:** 2023-04-18

**Authors:** Vignesh Venkatakrishnan, Jonas Elmwall, Trisha Lahiri, Martina Sundqvist, Linda Bergqvist, Hakon Leffler, Ulf J Nilsson, Amanda Welin, Johan Bylund, Anna Karlsson-Bengtsson

**Affiliations:** Department of Life Sciences, Chalmers University of Technology, 41296 Gothenburg, Sweden; Department of Rheumatology and Inflammation Research, Institute of Medicine, Sahlgrenska Academy, University of Gothenburg, 41756 Gothenburg, Sweden; Department of Rheumatology and Inflammation Research, Institute of Medicine, Sahlgrenska Academy, University of Gothenburg, 41756 Gothenburg, Sweden; Department of Rheumatology and Inflammation Research, Institute of Medicine, Sahlgrenska Academy, University of Gothenburg, 41756 Gothenburg, Sweden; Department of Rheumatology and Inflammation Research, Institute of Medicine, Sahlgrenska Academy, University of Gothenburg, 41756 Gothenburg, Sweden; Department of Rheumatology and Inflammation Research, Institute of Medicine, Sahlgrenska Academy, University of Gothenburg, 41756 Gothenburg, Sweden; Department of Laboratory Medicine, Lund University, 22100 Lund, Sweden; Centre for Analysis and Synthesis, Department of Chemistry, Lund University, 22100 Lund, Sweden; Division of Inflammation and Infection, Department of Biomedical and Clinical Sciences, Linköping University, 58183 Linköping, Sweden; Department of Oral Microbiology and Immunology, Institute of Odontology, Sahlgrenska Academy, University of Gothenburg, 41756 Gothenburg, Sweden; Department of Life Sciences, Chalmers University of Technology, 41296 Gothenburg, Sweden; Department of Rheumatology and Inflammation Research, Institute of Medicine, Sahlgrenska Academy, University of Gothenburg, 41756 Gothenburg, Sweden

**Keywords:** galectin-3, neutrophils, phagocytosis, reactive oxygen species, *Staphylococcus aureus*

## Abstract

Among the responders to microbial invasion, neutrophils represent the earliest and perhaps the most important immune cells that contribute to host defense with the primary role to kill invading microbes using a plethora of stored anti-microbial molecules. One such process is the production of reactive oxygen species (ROS) by the neutrophil enzyme complex NADPH-oxidase, which can be assembled and active either extracellularly or intracellularly in phagosomes (during phagocytosis) and/or granules (in the absence of phagocytosis). One soluble factor modulating the interplay between immune cells and microbes is galectin-3 (gal-3), a carbohydrate-binding protein that regulates a wide variety of neutrophil functions. Gal-3 has been shown to potentiate neutrophil interaction with bacteria, including *Staphylococcus aureus*, and is also a potent activator of the neutrophil respiratory burst, inducing large amounts of granule-localized ROS in primed cells. Herein, the role of gal-3 in regulating *S. aureus* phagocytosis *and S. aureus*-induced intracellular ROS was analyzed by imaging flow cytometry and luminol-based chemiluminescence, respectively. Although gal-3 did not interfere with *S. aureus* phagocytosis *per se*, it potently inhibited phagocytosis-induced intracellular ROS production. Using the gal-3 inhibitor GB0139 (TD139) and carbohydrate recognition domain of gal-3 (gal-3C), we found that the gal-3-induced inhibitory effect on ROS production was dependent on the carbohydrate recognition domain of the lectin. In summary, this is the first report of an inhibitory role of gal-3 in regulating phagocytosis-induced ROS production.

## Introduction

Galectin-3 (gal-3), a carbohydrate-binding protein with affinity for β-galactoside-containing glycoconjugates, is an emerging inflammatory modulator that is implicated in many physiological and pathological processes (Dumic et al. 2006; Henderson and Sethi 2009; Johannes et al. 2018). Of the 15 different galectins identified in mammals, gal-3 is the most widely studied, having significant roles in various diseases, including cancer (Newlaczyl and Yu 2011), fibrosis (Mackinnon et al. 2012; Slack et al. 2021), cardiovascular diseases (Suthahar et al. 2018), and microbial infections (Sehrawat and Kaur 2020). Gal-3 consists of a carbohydrate recognition domain (CRD; gal-3C) and an N-terminal collagen-like domain, which are responsible for glycan binding and the formation of lectin oligomers, respectively. The lectin can bind to glycosylated cell surface receptors and trigger or modulate cellular responses, such as cell adhesion, activation, and signaling (Almkvist and Karlsson 2002; Robinson et al. 2019).

Given its soluble nature and carbohydrate binding capability, gal-3 has been implicated as a critical immuno-modulatory molecule, especially with regards to neutrophil functions (Robinson et al. 2019). Neutrophils are central cells of the innate immune system, primarily dedicated to the killing of invading microbes by a process known as phagocytosis (Nauseef and Borregaard 2014; Burn et al. 2021). During phagocytosis, the microbe is engulfed into a phagosome that subsequently fuses with intracellular neutrophil granules (azurophil and specific granules) by which a plethora of proteolytic enzymes and other bactericidal molecules are released upon the internalized prey (Burn et al. 2021).

One of the important antimicrobial mechanisms in neutrophils is the production of reactive oxygen species (ROS) by the electron transporting enzyme NADPH-oxidase. This oxidase is assembled in neutrophil membranes upon cell activation (Babior 1999). As the membrane part of the enzyme is mainly present in specific granules, the fusion of specific granules with the phagosome membrane allows for the NADPH-oxidase to be assembled in the phagosome membrane, i.e. phagosome-localized intracellular ROS (icROS) is produced. With regards to gal-3, we have previously shown that this lectin directly induces icROS, but only in exudated or TNFα-primed neutrophils; resting blood neutrophils are inert to the lectin (Karlsson et al. 1998). The localization of the gal-3-induced icROS in primed or exudated cells has not yet been defined but we have suggested a nonphagosomal organelle formed by heterologous granule fusion as the site of activation (Bylund et al. 2010). Intracellular ROS production can be measured by MPO-dependent luminol-amplified chemiluminescence (CL), but there is no method to discriminate between the phagosome- and granule-localized icROS. The findings indicate that gal-3-induced neutrophil activation is restricted to sites of infection/inflammation where neutrophils are preactivated, or primed, a mechanism that may protect the host from collateral inflammatory damage.

Gal-3 has previously been shown to mediate neutrophil interaction with bacteria, including *S. aureus*, in an opsonin-like fashion (Farnworth et al. 2008; Quattroni et al. 2012; Bhaumik et al. 2013). Also, we have recently shown that gal-3 can play an important role in *S. aureus*-induced skin infection, where the lectin contributes to tissue damage induced by protease-expressing *S. aureus* (Elmwall et al. 2017). This inspired us to investigate the effect of gal-3 on the neutrophil interaction with *S. aureus*  *per se*, with a tentative hypothesis that gal-3 facilitates phagocytosis and participates in the host-pathogen interaction, possibly contributing to intraphagosomal ROS-dependent killing. For this purpose, we have used ROS-measurements to discern the effects of gal-3 on *S. aureus*-induced neutrophil NADPH-oxidase activation and phagocytosis. Our data, in opposite to our hypothesis, reveal a novel neutrophil-regulating function of gal-3, specifically inhibiting phagocytosis-induced icROS production, which is complementary to the previously described production of icROS in primed neutrophils triggered by gal-3 *per se*.

## Results

### Neutrophil intracellular ROS production is induced by serum-opsonized bacteria

**Fig. 1:**
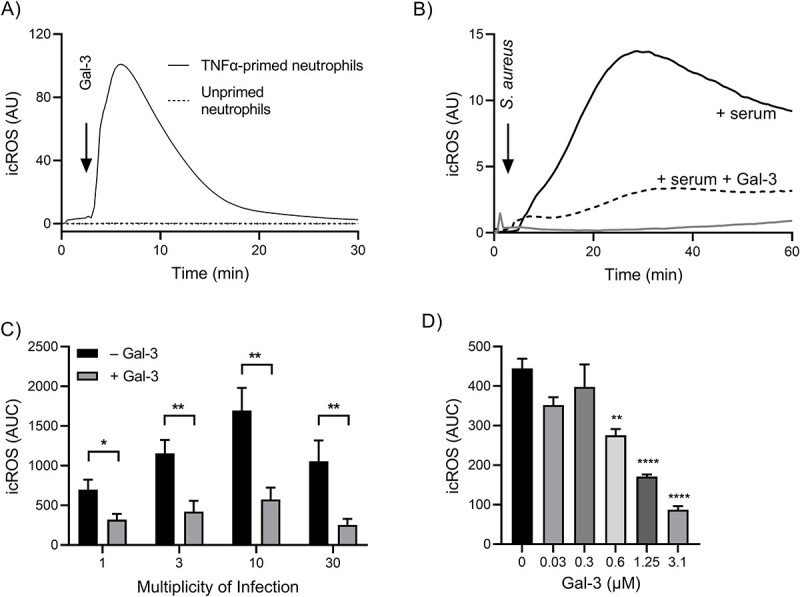
Gal-3 directly induces icROS production in TNFα primed neutrophils and inhibits phagocytosis-induced icROS production in neutrophils stimulated by *S. aureus*. The effect of gal-3 directly on neutrophils and on neutrophil interaction with *S. aureus* was investigated by measuring icROS production by luminol-amplified CL over time. (a) Gal-3 (1.25 μM) directly induces icROS production in TNFα primed (solid line) neutrophils and not in unprimed neutrophils (dotted line) and a time-trace of one representative experiment is shown (*n* = 4). (b) Suspensions of *S. aureus* were pretreated at 37°C for 30 min with and without gal-3 (1.25 μM) and/or 10% human serum and were then added to neutrophils at MOI 3:1 and time-trace of a representative phagocytosis-induced icROS production kinetics is shown (*n* = 4). The black solid line represents *S. aureus* and serum, the black dashed line represents *S. aureus*, serum, and gal-3, and the gray line represents only *S. aureus*. (c) the area under the curve values shown as mean ± SD (*n* = 4) of *S. aureus* induced icROS with and without gal-3 (1.25 μM) at different MOIs 1, 3, 10, and 30 per neutrophil. (d) The observed gal-3 induced icROS inhibition is concentration dependent shown in quantitative area under the curve analysis (mean ± SD), *n* = 4. The statistical analysis was performed using student’s *t*-test in (c) and one-way ANOVA followed by Dunnet’s multiple comparisons test in (d). ^*,^  ^*^^*^, and ^*^^*^^*^^*^ indicate *P*-values less than 0.05, 0.01, and 0.0001, respectively.

Intracellular ROS (icROS) can be generated either in the mature phagosome upon particle or microbe ingestion or, in the absence of phagosome formation, at another intracellular site hypothesized to be heterologously fused intracellular granules. As previously shown, gal-3 induced icROS production, measured using luminol-amplified CL, in TNFα-primed neutrophils, whereas unprimed resting neutrophils did not respond to the lectin (Fig. 1a). Hence, priming is a prerequisite for gal-3 activation of neutrophil icROS production. Hereafter, with regards to phagocytosis-induced icROS production, only unprimed, resting neutrophils were used in order to eliminate the contribution of gal-3-induced icROS to any measurements.

*S. aureus*-induced icROS production in unprimed neutrophils was assessed over time after addition of bacteria at a multiplicity of infection (MOI) i.e. microbes per neutrophils, of 3:1. As expected, unopsonized *S. aureus* induced very minor icROS production, whereas serum-opsonized *S. aureus* induced a pronounced response that was gradually increasing and long lasting (>1 h) (Fig. 1b). The kinetics of the response is typical for a respiratory burst associated with phagocytosis, suggesting that serum-opsonized *S. aureus* was phagocytized efficiently, whereas non-opsonized bacteria were not.

### Galectin-3 inhibits phagocytosis-induced icROS in a carbohydrate-dependent manner

To investigate whether gal-3 can influence the phagocytosis-induced icROS production, the lectin was added to the neutrophils together with *S. aureus* (MOI of 3:1) (Fig. 1b). Interestingly, the icROS-production in unprimed resting neutrophils was much lower in the presence of gal-3, i.e. gal-3 inhibited the phagocytosis-induced icROS (Fig. 1b). Neither serum, gal-3, nor serum and gal-3 by themselves gave any icROS-production in resting neutrophils (Supplementary Fig. S2). The phagocytosis-induced icROS in the absence of gal-3 was dose-dependent for MOIs between 1 and 30 (Fig. 1c) and statistically significant gal-3-induced inhibition was observed at all different MOIs. In subsequent experiments, an MOI of 3:1 was used to achieve a significant icROS response while avoiding the formation of large aggregates of cells and bacteria. The inhibition by gal-3 was concentration-dependent, present as low as 0.6 μM but more pronounced at 1.25 and 3.1 μM (Figs. 1d).

### Galectin-3-induced inhibition of phagocytosis-induced icROS is carbohydrate dependent

**Fig. 2:**
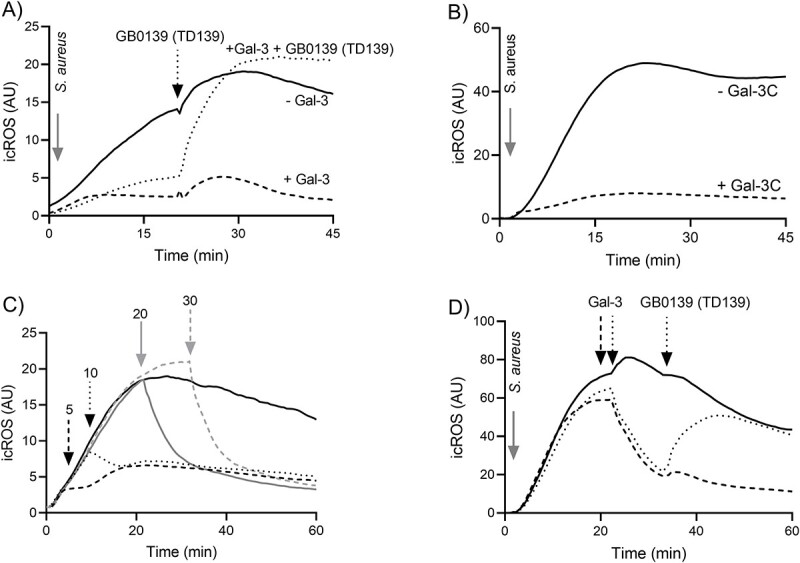
Gal-3 induced inhibition of phagocytosis-induced icROS is rapid and carbohydrate dependent. (a) Suspensions of *S. aureus* were pretreated at 37°C for 30 min with (dotted and dashed line) and without (full line) gal-3 (1.25 μM) were then added to neutrophils at MOI 3:1 and GB0139 (TD139) (gal-3 inhibitor, 10 μM) was added (indicated by an arrow) to one of the samples incubated with gal-3 (dotted line) during the run. Time-trace of a representative phagocytosis-induced icROS production kinetics is shown (*n* = 4). (b) Suspensions of *S. aureus* were pretreated at 37°C for 30 min with and without gal-3C (1.25 μM) were then added to neutrophils at MOI 3:1, and icROS production was measured by luminol-amplified CL over time and time-trace of one representative experiment is shown (*n* = 4) (c) The effect of gal-3 on an on-going neutrophil respiratory burst response to *S. aureus* was investigated by measuring icROS production by luminol-amplified CL over time. Suspensions of serum opsonized *S. aureus* (MOI 3:1) were added as stimuli at 0 min to neutrophils and gal-3 (1.25 μM) added to the suspension at different time points indicated by arrows. The addition of gal-3 at the later time points induced faster inhibition as evident from the representative (*n* = 4) time-trace kinetics. (d) Suspensions of *S. aureus* were added to neutrophils at MOI 3:1 and gal-3 (1.25 μM) was added during the run (indicated by an arrow) to two of the samples (dotted and dashed line indicated by arrow) and GB0139 (TD139) (gal-3 inhibitor, 10 μM) was added (indicated by an arrow) to one of the samples incubated with gal-3 (dotted line) during the run. Time-trace of a representative phagocytosis-induced icROS production kinetics is shown (*n* = 4).

Since gal-3 is a carbohydrate-binding protein, we then investigated the role of carbohydrates in mediating the interaction and inhibitory mechanism. Using GB0139 (TD139), a synthetic gal-3 inhibitor blocking the carbohydrate recognition domain of gal-3 (Delaine et al. 2016; Humphries et al. 2022), gal-3-induced icROS inhibition was recovered (Fig. 2a). The inhibition of phagocytosis-induced icROS was not only mediated by gal-3, but also by gal-3C (Fig. 2b), a truncated gal-3 containing only the carbohydrate recognition domain and not the N-terminal domain. This shows that oligomerization of gal-3 occurring through the N-terminal domain (type-N self-association) is not necessary for the inhibitory effect as it can also be achieved using gal-3C, which can form both heterologous and homologous oligomers, through type-C self-association (Sundqvist et al. 2018). Together, these data strongly suggest a critical role played by carbohydrate-containing surface epitopes for the inhibitory effect.

### Galectin-3 induces rapid termination of phagocytosis-induced icROS

To better understand the inhibitory mechanism mediated by gal-3 on the *S. aureus*-induced icROS production in neutrophils, we further investigated the importance of preincubation of bacteria with gal-3. Instead of adding gal-3 together with *S. aureus*, the lectin was added to the neutrophils in the luminometer, at different time points (5, 10, 20, and 30 min) during an on-going respiratory burst response. Although, at all time-points, the ongoing activity was inhibited by gal-3, at 20 and 30 min into the response the inhibition was rapid and the response terminated quickly (Fig. 2c). These results indicate that gal-3 interacts with the neutrophils per se and not with the (at this point phagocytosed) bacteria, potentially inducing an inhibitory signal that blocks further activation of NADPH-oxidase by the phagocytic process. Inhibition of phagocytosis-induced icROS when gal-3 was added during on-going measurement, was also recovered using GB0139 (TD139) (Fig. 2d). The findings strongly suggest that the inhibition mechanism is mediated by carbohydrate-containing surface molecules and that neutrophil interaction is critical for the inhibitory effect of gal-3.

### Inhibition of *S. aureus*-induced icROS is specific to galectin-3

**Fig. 3:**
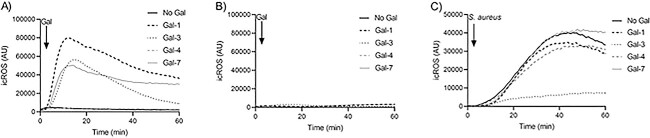
Inhibition of *S. aureus*-induced icROS is specific to galectin-3. (a–b) Gal-1 and -7 directly induces icROS in TNFα primed neutrophils (a) and not in unprimed neutrophils (b) at the same concentration (1.25 μM) as gal-3, whereas gal-4 did not induce any icROS production (*n* = 4) measured using luminol-amplified CL over times. (c) Suspensions of serum opsonized *S. aureus* were pretreated with and without 1.25 μM gal-1, -3 , -4, and -7 at 37°C for 30 min prior addition to neutrophils at MOI 3:1 and measurement of phagocytosis-induces icROS production over time. Time-trace of one representative experiment is shown (*n* = 4), which demonstrates that unlike gal-3, none of the other galectins inhibited the *S. aureus*-induced icROS production.

We also tested the capacity of Gal-1, 4, and 7 in inducing direct activation of icROS in resting and TNFα-primed neutrophils and their role in phagocytosis-induced icROS. These were chosen based on differences in lectin structure. Unlike Gal-3, which is a chimera-type galectin, Gal-1 and -7 belong to the prototypical subfamily with one carbohydrate recognition domain (CRD) forming a homodimer, whereas Gal-4 belongs to the tandem repeat subfamily, which contains two CRDs in tandem joined by a linker region (Johannes et al. 2018). Although none of the galectins induced icROS in resting neutrophils, Gal-1 and -7 directly induced icROS in TNFα-primed cells just as Gal-3, at the same concentration (1.25 μM) (Fig. 3a and 3b). Gal-4 did not induce any ROS production. To test the effect on *S. aureus*-induced icROS, we preincubated neutrophils with the galectins (1.25 μM) before stimulating with *S. aureus* and measured the production of icROS. In contrast to gal-3 and -3C, none of the other galectins inhibited phagocytosis-induced icROS (Fig. 3c). Thus, the icROS-inhibitory effect is specific to gal-3, more specifically to the lectin part of gal-3 i.e., gal-3C.

### Galectin-3-induced inhibition of phagocytosis-induced icROS production is not specific to *S. aureus*

**Fig. 4:**
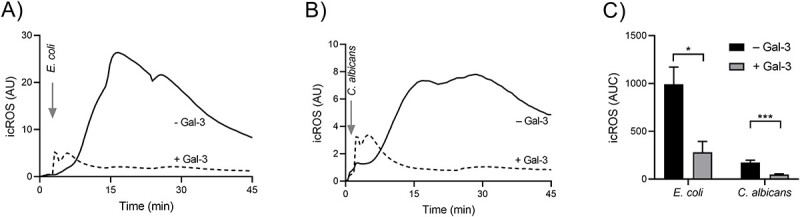
Gal-3 induced inhibition is not specific to *S. aureus.* The effect of gal-3 (1.25 μM) on neutrophil interaction with (a) *E. coli* (MOI 3:1) and (b) *C. albicans* (MOI 1:1) was investigated by measuring icROS production by luminol-amplified CL, and time-trace of a representative experiment (*n* = 4) is shown. The corresponding quantitative area under the curve values are shown as mean ± SD (*n* = 4) in (c). It is quite evident that gal-3 inhibition of phagocytosis induced icROS is not specific to gram-positive bacteria (*S. aureus*) but also to gram-negative *E. coli* and fungi. The statistical analysis in (c) was performed using student’s *t*-test and ^*^ and ^*^^*^^*^ indicate *P*-values less than 0.05 and 0.001, respectively.

Neutrophil phagocytosis can be directed by different mechanisms, depending on the identity of the prey. We wanted to investigate whether the gal-3-mediated inhibition of *S. aureus*-induced icROS is a more general mechanism applying to several microbes. Similar inhibitory effects were observed for both *E. coli* (gram negative bacteria) and *C. albicans* (yeast) (Fig. 4a–c), suggesting that the gal-3-dependent inhibition of phagocytosis-induced icROS is not specific to *S. aureus* but a more general phenomenon.

### Galectin-3 does not inhibit neutrophil uptake of *S. aureus* but may influence bacterial killing

**Fig. 5:**
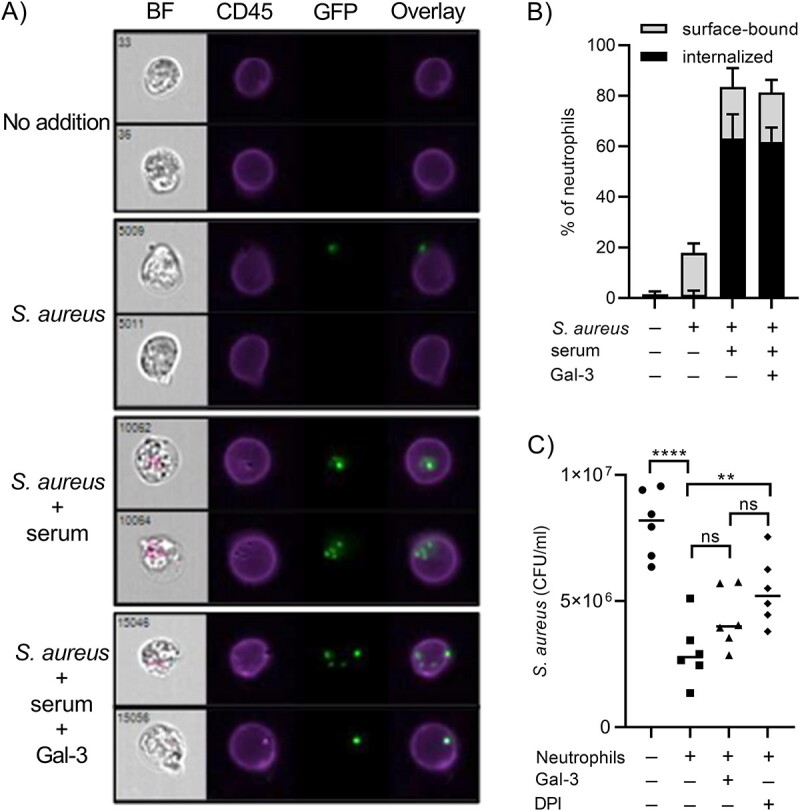
Gal-3 has no influence on attachment or phagocytosis and killing of serum-opsonized *S. aureus*. Neutrophils were incubated with GFP-expressing *S. aureus* pre-treated with and without serum (10%) and gal-3 (1.25 μM) at MOI 3:1 for 30 min at 37°C, after which the cells were fixated with paraformaldehyde (2%) and surface stained with Alexa flour 405-conjugated CD45 antibody. In (a), representative bright-field (BF) and fluorescence images (CD45, neutrophil cell surface staining; GFP, *S. aureus*) as well as fluorescence image overlays are shown. Bacteria were detected both within and attached to the neutrophils, and no qualitative differences could be seen in the presence or absence of gal-3. This was corroborated with quantitative analysis shown as mean ± SD (*n* = 4) percentage of neutrophils with associated GFP signal, calculated from imaging flow cytometry-based quantification of surface-bound and internalized *S. aureus*. The gating strategy employed in quantitative analysis is given in Supplementary Fig. S1. (c) Neutrophils were incubated with *S. aureus* in the presence and absence of gal-3 (1.25 μM) or diphenyleneiodonium chloride (DPI, 10 μM) for 45 min at 37°C and plated on LB-agar and incubated overnight at 37°C. The number of colonies were counted and CFU/ml was calculated and represented. DPI was used as NADPH oxidase inhibitor control, and at 10 μM, DPI completely abolishes icROS. Gal-3 tends to be involved in *S. aureus* killing, although not statistically significant. Data are represented as mean ± SEM, and the statistical analysis in (c) was performed using one-way ANOVA followed by Tukey’s multiple comparisons test. ^*^^*^ and ^*^^*^^*^^*^ indicate *P*-values less than 0.01 and 0.0001, respectively.

The most obvious question invoked by the gal-3 inhibition of *S. aureus*-induced icROS is whether gal-3 inhibits the uptake of the *S. aureus* bacteria into phagosomes, thereby also inhibiting the resulting respiratory burst. We therefore tested the effect of gal-3 on neutrophil adhesion to and uptake of serum-opsonized GFP-labeled *S. aureus*, using imaging flow cytometry (ImageStreamX). A significant quantity of bacteria was seen on and within the neutrophil perimeter after 30 min of co-incubation (Fig. 5a and b), showing that the bacteria were attached to as well as phagocytosed by the neutrophils. Surprisingly, the addition of gal-3 during incubation of neutrophils with *S. aureus* did not in any way affect neither attachment nor uptake of the bacteria into the cells. This suggests that the gal-3-induced inhibition of phagocytosis-dependent icROS-production was not due to inhibition of interaction between the bacteria and the neutrophils *per se*.

Subsequently, we wanted to investigate whether gal-3 inhibition of icROS affects the killing of *S. aureus* by neutrophils using colony forming unit (CFU) based growth assay (Fig. 5c). First, we established that icROS affects *S. aureus* killing by using diphenyleneiodonium (DPI), a potent NADPH-oxidase inhibitor that completely abolishes icROS production (Buck et al. 2019). Here, DPI significantly inhibited bacterial killing (Fig. 5c), establishing the connection between the two parameters. Addition of gal-3 to the neutrophil/*S. aureus* co-incubation resulted in a reduction of live bacteria in each experiment. Although there is a trend toward inhibition of *S. aureus* killing by gal-3, there was no statistically significant difference between the groups. One possible explanation to the lesser effect of gal-3 as compared to DPI is that gal-3 does not completely inhibit phagocytosis-induced icROS while DPI is a potent icROS inhibitor. In conclusion, gal-3 does not inhibit attachment or uptake of *S. aureus* but may possibly affect killing the bacteria through inhibiting NADPH-oxidase-derived icROS.

## Discussion

In this study, we have investigated the effect of gal-3 during neutrophil phagocytosis and found a novel inhibitory role of gal-3 on phagocytosis-induced icROS. This is in contrast to its ability to directly induce icROS production in primed neutrophils (Karlsson et al. 1998). Our tentative hypothesis was that gal-3 would interact within the binding of the neutrophil to the bacterium and thereby interfere with the phagocytic process. The opposite outcome, that gal-3 would contribute to the uptake and internalization of the bacteria, was also a possibility, as we have previously shown gal-3 to function as opsonin in clearance of apoptotic neutrophils by macrophages (Karlsson et al. 2009) and as gal-3 is well known to interact with bacteria (Gupta et al. 1997; Fowler et al. 2006; Elmwall et al. 2017). Surprisingly, inhibition of the *S. aureus*-induced icROS response was instead observed, which makes gal-3 a unique molecule by directly inducing icROS as a soluble stimulus and, at the same time, inhibiting phagocytosis-induced icROS, a finding that, to our knowledge, has not been shown before. Taken together, the data suggest that in the absence of a phagosome, gal-3 directly activates non-phagosomal (probably granule-localized) icROS, whereas, during phagocytosis, it inhibits phagocytosis-induced icROS. The mechanism behind gal-3-induced inhibition, i.e. by which mechanisms the inhibition occurs, is yet to be investigated. On a methodological note, as neither chemiluminescence nor other techniques that measure icROS cannot differentiate between granule- and phagosome-localized icROS (Bylund et al. 2010; Dahlgren et al. 2019), there is an urgent need to develop a method that can distinguish the two.

The initiation of *S. aureus* phagocytosis by neutrophils requires bacterial surfaces decorated with opsonins, e.g. immunoglobulins or complement, that are recognized by neutrophil Fc- and/or complement receptors (van Kessel et al. 2014). To investigate the involvement of complement in our experimental setup, we opsonized *S. aureus* with heat-inactivated serum. We found a substantial decrease in phagocytosis-induced icROS, concluding that phagocytosis and the subsequent generation of icROS was primarily complement dependent (Supplementary Fig S3). The complement receptor 3 (CR3) has been shown to be involved in gal-3-induced effects in other settings. Wu et al suggest that endogenous galectin-3 attenuates ROS-dependent killing of Candida in neutrophils, mediated by endogenous, cytosolic gal-3 affecting the CR3 downstream signaling pathway (including that leading to ROS generation; Wu et al. 2017). As we have used exogenous gal-3, the interaction inducing the functional effects shown here is likely to be on the cell surface. If CR3 is involved, a speculation would be that gal-3 crosslinks CR3 (and/or other CRs) with other receptors, inducing a signaling cascade that inhibits phagocytosis-induced icROS.

The inhibitory effect of gal-3 on the phagocytosis induced icROS was shown to be carbohydrate-dependent, both by using a lactose analogue as well as truncated galectin-3, gal-3C, containing only the CRD. Specific functional glycoconjugate receptors, glycoproteins and/or glycolipids, for gal-3-induced downstream signals in neutrophils remain largely unknown. In our recent glycomic characterization of neutrophil granules and plasma membrane proteins, we found unusually elongated polylactosamine-containing glycans in the membranes of the specific and gelatinase granules, whereas the cell surface was shown to be covered with sialylated polylactosamine glycans (Venkatakrishnan et al. 2020). Gal-3 was shown to have affinity toward these polylactosamine structures that also were shown to be critical for enhanced respiratory burst response in primed neutrophils (Salomonsson et al. 2010). CD66a and CD66b have previously been characterized as prominent receptor candidates for gal-3 binding in neutrophils (Stocks et al. 1996; Feuk-Lagerstedt et al. 1999) but, as stated above, CR3 is also a potential receptor candidate together with other neutrophil glycoconjugates. With regards to lactose-containing glycosphingolipids, we and others have characterized the lipid composition of neutrophil granules and plasma membrane and found the presence of lactosyl ceramide, whose function remains unknown (Symington et al. 1987; Karlsson et al. 2001).

Considering the fact that the glycosylated receptor(s) are localized intragranularly and that degranulation of neutrophils is needed to mobilize them onto the cell surface, granule mobilization is a critical process for gal-3-mediated regulation of neutrophil functions. From this we can also draw the conclusion that degranulation and receptor mobilization to the cell surface most probably takes place in parallel to the phagocytosis process, upregulating the receptors and gal-3 through its interaction with the surface glycoconjugate receptor mediate the icROS inhibition induced by gal-3. Comprehensive characterization of gal-3-binding receptors in neutrophils is the first step toward understanding the regulatory function that gal-3 exerts on neutrophils and to determine whether the same or different glycoconjugate receptors are involved in activation and inhibition of ROS.

The fact that phagocytosis-induced icROS could be inhibited without affecting phagocytic uptake/killing of bacteria indicates that the signaling pathways that lead to NADPH-oxidase activation in the phagosomal compartment might differ from the signaling pathways that drive the phagosome formation. Interestingly, our data, although not statistically significant, indicate that gal-3-mediated inhibition of phagocytosis-induced icROS can be associated with inhibited bacterial killing. Respiratory burst is one of the essential killing mechanisms deployed by neutrophils and its importance is exemplified by chronic granulomatous disease (CGD) neutrophils (Marciano et al. 2015), which do not produce ROS and show impaired microbial killing, in line with our data.

It is not necessarily so that icROS production induced by microbial ingestion (phagocytosis) is exclusively localized in the phagosome, neither *in vivo* nor *in vitro*. In some experiments where gal-3 was added together with the microbes to unprimed resting neutrophils, there was a small icROS response during the first 10 min of the measurement (Fig. 1b). It could be speculated that this separate and small response is due to direct activation of granule-localized icROS in unprimed neutrophils by gal-3 as a result of phagocytosis-induced upregulation to the cell surface of activating gal-3 receptors. This together suggests that gal-3 specifically inhibits the phagosome-localized icROS, potentially by inhibiting signals leading up to the direct activation of the NADPH-oxidase.

The functional aspects of the gal-3-dependent inhibition of phagocytosis-induced icROS production are intriguing. Gal-3 is ubiquitously expressed in adults, found in many normal tissues and in areas of inflammation, including cancer tissues (Idikio et al. 1998; Hsu et al. 1999; Kawachi et al. 2000; Newlaczyl and Yu 2011). Turning off the neutrophil respiratory burst could be part of a tissue protective act, avoiding leakage of tissue-destructive ROS in a situation where white blood cells are in the midst of a fulminant infection. Another angle would be that *S. aureus*, and perhaps other pathogens, utilize the inhibitory effect of gal-3 to protect themselves from the bactericidal ROS within the phagosome, by inducing tissue production of gal-3 to inhibit the phagocytosis-induced NADPH-oxidase activation. Gal-3 inhibition of phagocytic ROS production as a virulence mechanism for *S. aureus* has thus to be considered in future studies. Such a mechanism could be critical in acute infectious or inflammatory diseases such as sepsis, where the soluble level of gal-3 is upregulated (Mishra et al. 2013; Kim et al. 2017) and neutrophil responses are of the essence (Farnworth et al. 2008; Ferreira et al. 2018).

In summary, the data presented here indicate a role of gal-3 as a potent inhibitor of phagocytosis-induced icROS, apart from being a potent activator of icROS in the absence of phagocytosis. The exact molecular mechanism and the signaling pathway involved in gal-3 inhibition of phagocytosis-induced icROS remains to be investigated. This novel inhibitory role played by gal-3 during phagocytosis is carbohydrate-dependent and the inhibition could possibly regulate bacterial killing. A dual role for gal-3 in regulating neutrophil functions could potentially be exploited as one of the strategies to augment first line of host defense against severe infections.

## Materials and methods

### Isolation of neutrophils

Neutrophils from buffy-coats (obtained from the blood bank at Sahlgrenska University Hospital) were isolated by dextran sedimentation and Ficoll-Paque (Cytiva, GE-Healthcare Bioscience) separation (Boyum et al. 1991). Remaining erythrocytes were removed by hypotonic lysis and isolated neutrophils were resuspended in Krebs Ringer phosphate buffer supplemented with Ca^2+^ (1 mM).

### Microbial culture

*Staphylococcus aureus* laboratory strain 8325-4 transformed with plasmid pCM29 for green fluorescent protein (GFP) expression and chloramphenicol resistance was used in this study. Batches of *S. aureus* were prepared as follows: *S. aureus* 8325-4 was inoculated in tryptic soy broth (TSB) supplemented with 10 μg/ml chloramphenicol, cultured overnight at 37°C. *Escherichia coli* strain DH12α was grown on Luria-Bertani (LB) agar at 37°C, overnight and single colonies were added to LB broth, cultured overnight at 37°C*. Candida albicans* strain sc5314 was used for the preparation of yeast. The fungus was cultured on Sabouraud dextrose agar plates for 24 h at 37°C in an aerobic environment and four to five distinct colonies were added to 5 ml RPMI media for 24 h at 30°C with little rotation.

The GFP labeled bacteria and *C. albicans* were washed three times, diluted in Krebs-Ringer phosphate buffered saline (KRG; pH 7.3) with 1 mM Ca^2+^ and enumerated using an Accuri C6 flow cytometer (Becton Dickinson, San Jose, CA, USA), and then kept frozen at −80°C until use.

### Purification of galectin-3

Recombinant human gal-3 was produced in *E. coli* and purified as described (Massa et al. 1993). After isolation, gal-3 was kept at 4°C in phosphate-buffered saline (PBS; pH 7.2) containing lactose (150 mM) until applied to a PD-10 desalting gel-filtration column (Cytiva, GE-Healthcare Bioscience) to remove lactose. The protein concentration was measured using Micro BCA™ protein assay kit (Thermo Fisher Scientific) and diluted in KRG (pH 7.3) supplemented with Ca^2+^ (1 mM), and stored at −80°C.

### Luminol-amplified chemiluminescence

Intracellular ROS (icROS) generated by the neutrophil NADPH-oxidase was determined by luminol-amplified chemiluminescence (CL; Bylund et al. 2014; Dahlgren et al. 2020) in a six-channel Biolumat LB 9505 (Berthold Technologies, Bad Wildbad, Germany). Luminol (50 μM, Sigma) was added together with the extracellular radical scavengers SOD (50 U/ml; Worthington) and catalase (1000 U/ml; Worthington) to measure specifically intracellular NADPH-oxidase activity. If not stated otherwise, cells were equilibrated for five min at 37°C before the stimuli, serum opsonized (10%) or unopsonized *S. aureus, E. coli*, or *C. albicans* (at a multiplicity of infection (MOI) i.e. microbes per neutrophils as stated in each figure legend) were added, and CL was recorded over 30–60 min. Galectins (gal-1, -3, -4, -7, -3C) were either included during opsonization of stimuli or added to the neutrophil—bacteria sample during the measurement, as stated in each figure legend. When the gal-3 inhibitor GB0139(TD139) was used, this was added during measurements to a final concentration of 10 μM.

### Phagocytosis of *S. aureus*

To investigate the effect of gal-3 on neutrophil attachment and uptake of *S. aureus*, GFP-labeled *S. aureus* and neutrophils were incubated at an MOI of 3:1 at 37°C for 30 min with and without gal-3 (1.25 μM). Cells were then fixed with 2% paraformaldehyde (Roche Diagnostics) for 20 min while kept on ice. The cells were centrifuged at 300 g, washed once in PBS and then stained with Alexa Fluor 405–conjugated mouse IgG anti-human CD45 antibody (Invitrogen) for 30 min at 4°C. The samples were washed and resuspended in PBS and analyzed using an ImageStreamX Mark II imaging flow cytometer (Amnis, Seattle, WA, USA). The imaging flow cytometry analysis delivers bright field images and images with the respective florescent probe (CD45 and GFP). The images were further analyzed using IDEAS 6.2 software, employing the gating strategy described in Supplementary Fig. S1.

### *S. aureus* killing assay

To investigate the role of gal-3 on the ability of neutrophils to kill the bacteria, *S. aureus* and neutrophils (MOI 10:1) were incubated at 37°C for 45 min with and without gal-3 (1.25 μM) or 10 μM diphenyleneiodonium (DPI; Sigma). Post incubation, 0.01% saponin (Sigma) was added to the mix to briefly lyse neutrophils, samples were vortexed and serially diluted using sterile PBS and plated on LB-agar. The plates were incubated overnight at 37°C and the colonies were counted manually to determine CFU/ml.

### Statistical analysis

Statistical analysis was performed using GraphPad Prism v.9.01 (GraphPad Software, San Diego, CA, USA). Data represented as either mean ± SD or mean ± SEM and a Student’s *t*-test and one-way ANOVA followed by either Dunnet’s or Tukey’s multiple comparisons test was used for comparisons of datasets.

## Supplementary Material

Supplementary_File_cwad032

Supplementary_File_with_an_added_new_figure_cwad032

## Abbreviations

CL, chemiluminescence; DPI, diphenyleneiodonium; Gal, galectin; icROS, intracellular-reactive oxygen species; NADPH-oxidase, nicotinamide adenine dinucleotide phosphate-oxidase
