# Supplementary material for: Novel inhibitory effect of galectin-3 on the respiratory burst induced by Staphylococcus aureus in human neutrophils
Source: Glycobiology. 2023 Apr 18;33(6):503–11. doi: 10.1093/glycob/cwad032 (PMC10284110; doi:10.1093/glycob/cwad032)

**Supplementary File**

**Novel inhibitory effect of galectin-3 on the respiratory burst induced by *Staphylococcus aureus* in human neutrophils**

Vignesh Venkatakrishnan^1,2*^, Jonas Elmwall^2^, Trisha Lahiri^2^, Martina Sundqvist^2^, Linda Bergqvist^2^, Hakon Leffler^3^, Ulf J. Nilsson^4^, Amanda Welin^5^, Johan Bylund^6^, Anna Karlsson-Bengtsson^1,2^

^1^Department of Biology and Biological Engineering, Chalmers University of Technology, Gothenburg, Sweden

^2^Department of Rheumatology and Inflammation Research, Institute of Medicine, Sahlgrenska Academy, University of Gothenburg, Gothenburg, Sweden

^3^Department of Laboratory Medicine, Lund University, Lund, Sweden

^4^Centre for Analysis and Synthesis, Department of Chemistry, Lund University, Lund, Sweden

^5^Division of Inflammation and Infection, Department of Biomedical and Clinical Sciences, Linköping University, Linköping, Sweden

^6^Department of Oral Microbiology and Immunology, Institute of Odontology, Sahlgrenska Academy, University of Gothenburg, Gothenburg, Sweden

***Corresponding author**

Dr. Vignesh Venkatakrishnan

Department of Biology and Biological Engineering

Chalmers University of Technology

Gothenburg, Sweden

Email: vignesh.venkatakrishnan@chalmers.se

Tel: +46 736 43 16 01

**Supplementary Figure S1. Strategy of gating and analysis of imaging flow cytometry data for enumeration of neutrophils with attached and phagocytosed *S. aureus***. Neutrophils were incubated with serum-opsonized GFP-expressing *S. aureus* (MOI 3) with or without gal-3 for 15 min at 37°C, after which the cells were fixed with paraformaldehyde and surface stained with Alexa Fluor 405-conjugated CD45 antibody. Images were acquired in the imaging flow cytometer. A gating strategy was developed using all events from all samples. After color compensation, analysis in IDEAS software was performed by gating populations as follows: A) in-focus cells, as determined by the gradient RMS (root mean square for image sharpness) on the bright-field images; B) single cells, estimated by an object area and aspect ratio in bright-field images; C) neutrophils, identified by side-scatter and CD45-intensity ; D) bacteria-positive neutrophils, determined by raw max pixel and intensity of the GFP-signal; E) discriminating intracellular bacteria within the bacteria-positive neutrophil population by an adapted feature. First, the intracellular region was determined based on the CD45 signal image, eroded to exclude any surface-bound bacteria. Second, an intensity threshold was applied, to exclude signal artefacts from unfocused bacteria. Finally, spots of individual bacteria were identified by GFP signal peaks and counted using the built-in spot count function in IDEAS software. Exact definition on the spot count feature is in panel E. Gating of objects using this feature allowed for identification of neutrophils with internalized *S. aureus*.

**Supplementary Figure S2.** Serum, gal-3 and combination of the two do not induce icROS production similar to that of phagocytosis-induced.

**Supplementary Figure S1**


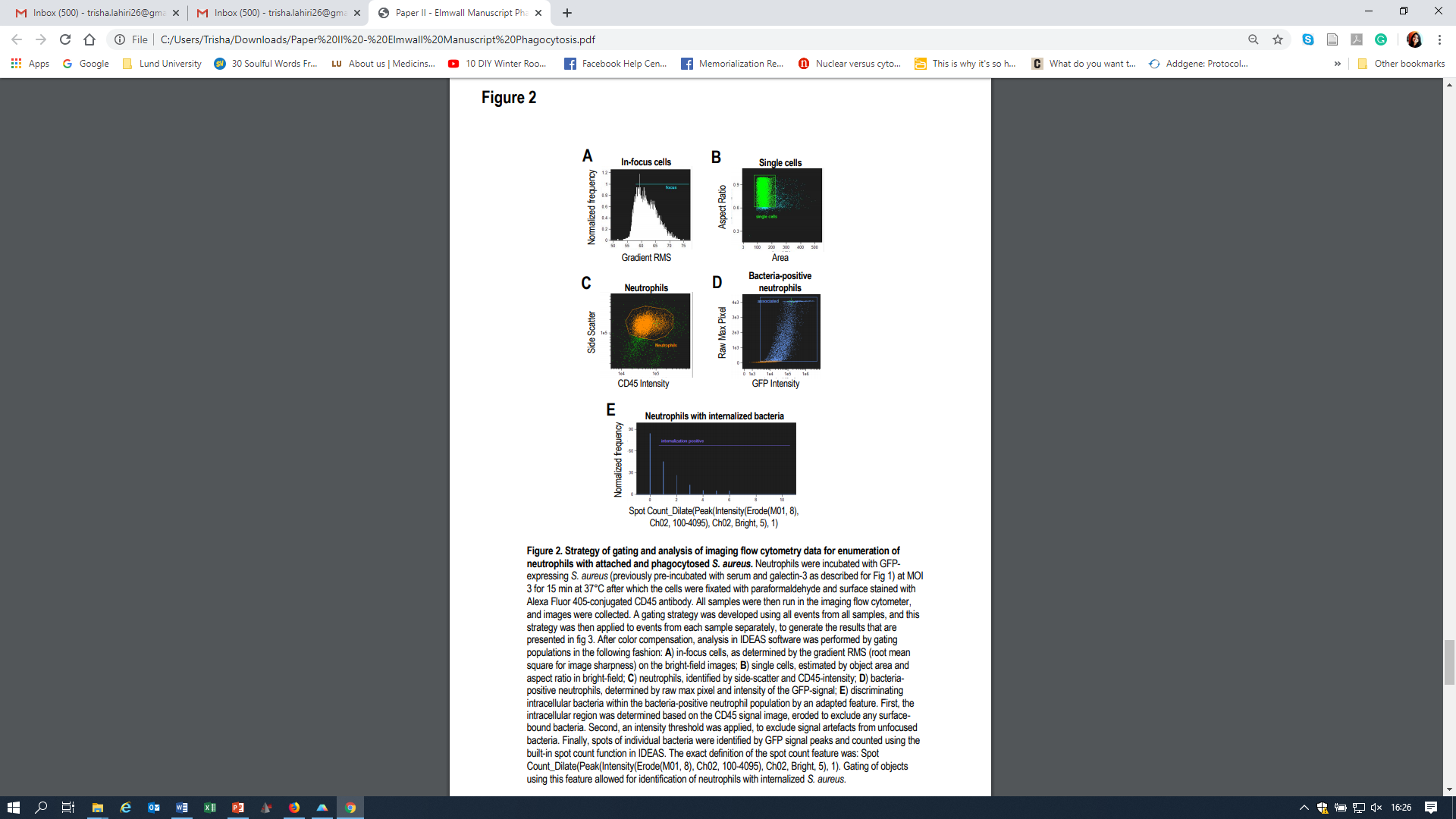


**Supplementary Figure S2**


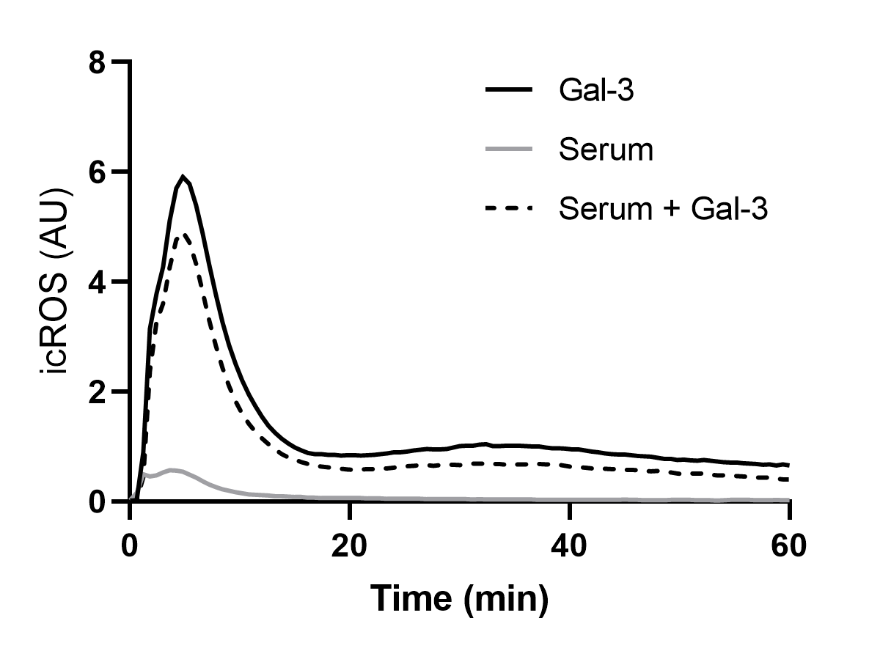

Supplement: Supplementary_File_cwad032 [file supplementary_file_cwad032.docx]
